# Saliva-derived exosomes regulate fibroblast metabolic reprogramming in skin wound healing

**DOI:** 10.3389/fcell.2025.1606716

**Published:** 2025-07-23

**Authors:** Sijia Song, Rong Xiang, Siyu Chen, Jianbo Wu, Wenxia Chen, Xianyu Li

**Affiliations:** ^1^Department of Operative Dentistry and Endodontology, College of Stomatology, Hospital of Stomatology, Guangxi Medical University, Nanning, China; ^2^ Guangxi Health Commission Key Laboratory of Prevention and Treatment for Oral Infectious Diseases, Nanning, China; ^3^ Guangxi Key Laboratory of Oral and Maxillofacial Rehabilitation and Reconstruction, Nanning, China; ^4^ Guangxi Clinical Research Center for Craniofacial Deformity, Nanning, China

**Keywords:** saliva-derived exosomes, glycolysis, metabolic reprogramming, wound healing, extracellular matrix remodeling, fibroblasts

## Abstract

**Background:**

Effective skin repair requires rapid wound closure accompanied by precise extracellular matrix (ECM) remodeling and balanced cellular metabolism. Saliva-derived exosomes (S-Exo) have emerged as promising therapeutic agents due to their rich bioactive components; however, their mechanisms in ECM remodeling and metabolic regulation remain unclear. This study aimed to elucidate how S-Exo modulate ECM turnover through metabolic reprogramming, particularly glycolysis, in human skin fibroblasts (HSFs), and identify critical exosomal molecules mediating these effects.

**Methods:**

S-Exo were isolated and characterized. A rat full-thickness skin defect model and *in vitro* assays with human skin fibroblasts and HaCaT keratinocytes were employed to evaluate S-Exo effects on wound closure, ECM remodeling, and cellular metabolism. Transcriptomic profiling of wound tissues, targeted metabolomic analysis of fibroblasts, and proteomic evaluation of S-Exo cargo were performed to explore underlying mechanisms. Metabolic interventions further confirmed the contribution of metabolic modulation to S-Exo-mediated wound healing.

**Results:**

S-Exo significantly accelerated wound healing by enhancing fibroblast viability, migration, and ECM remodeling, characterized by elevated secretion of matrix metalloproteinases (MMP1 and MMP3). Transcriptomic, metabolomic, and proteomic analyses revealed that S-Exo robustly activated key metabolic pathways, particularly glycolysis, reflected by increased expression of glycolytic genes (e.g., GLUT1, HK2, PFKM) and enhanced glycolytic flux in fibroblasts. Remarkably, S-Exo were found to carry nearly all enzymes involved in glycolysis, indicating an underlying enzyme-transfer mechanism for sustained metabolic modulation. Importantly, glycolytic activity positively correlated with MMP secretion, and inhibition of glycolysis significantly reduced MMP production, highlighting glycolysis as a crucial regulator of ECM remodeling.

**Conclusion:**

Saliva-derived exosomes promote wound healing by potentially modulating fibroblast metabolism via exosome-associated glycolytic enzymes, enhancing glycolytic flux, and thereby regulating ECM remodeling via increased MMP secretion. These findings provide novel insights into metabolism-targeted exosome therapies for wound healing.

## Graphical Abstract

**Figure:**
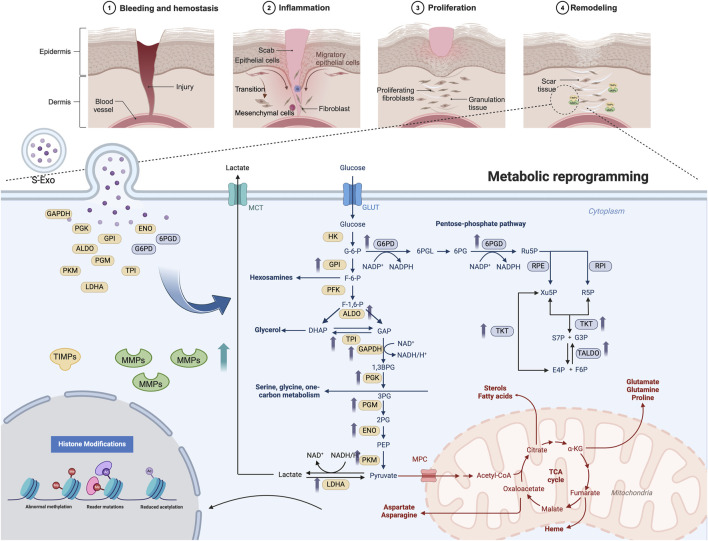
S-Exos promote wound healing by delivering key metabolic enzymes to recipient cells, triggering metabolic reprogramming and enhancing MMP secretion. This process facilitates ECM remodeling, accelerating tissue repair and reducing fibrosis. The top panel illustrates wound healing stages, while the lower panel highlights S-Exo-induced metabolic shifts. Created in BioRender. Song, S. (2025) https://BioRender.com/w32q619.

## Introduction

Skin wound repair is a highly energy-dependent biological process (Woo et al., 2023). It involves the coordinated action of various cell types and complex molecular mechanisms, all of which are intricately regulated to restore tissue integrity. A successful healing process demands two key outcomes: rapid wound closure and minimal scar formation (Baron et al., 2020). The repair process requires rapid cell proliferation, migration, and synthesis of extracellular matrix (ECM), which places a high demand on energy and biosynthetic precursors (Demling, 2009). Efficient metabolic pathways are essential for generating ATP and providing the building blocks for proteins, lipids, and nucleic acids, thereby supporting the cellular functions necessary for tissue repair (Wang et al., 2024).

Recent studies have highlighted the critical role of metabolic reprogramming during wound healing, particularly the shift towards enhanced glycolysis. This shift, commonly referred to as the Warburg effect, is frequently observed in cells with high proliferative potential, such as regenerating stem cells and cancer cells, where glycolysis becomes the predominant pathway for energy production (Vaupel and Multhoff, 2021). Accumulating evidence indicates that aerobic glycolysis exerts a more pronounced impact on wound closure compared to oxidative phosphorylation (OXPHOS) (Manchanda et al., 2023). In mammalian skin, enhancing glycolysis via pyruvate dehydrogenase kinase 4 (PDK4) or treatment with enhanced platelet-rich plasma (ePRP) significantly alleviates fibroblast senescence, restores their proliferative and migratory capabilities, reduces intracellular ROS levels, and accelerates wound repair through metabolic reprogramming (Ma Z. et al., 2023; Weng et al., 2021). Similarly, in hair follicle stem cells, LSD1 associates with HSP90 to stabilize c-MYC, thereby increasing LDHA expression and glycolysis, subsequently promoting stem cell proliferation, differentiation, and skin regeneration (Li et al., 2023). Effective wound healing requires rapid closure, functional restoration, and minimal scar formation, thus preserving tissue integrity and aesthetic outcomes (Talbott et al., 2022).

Over the past decade, exosomes have emerged as critical mediators of intercellular communication, demonstrating considerable potential in regenerative medicine, particularly through their ability to modulate cellular metabolism and enhance tissue repair processes (Isaac et al., 2021). Growing evidence indicates that exosomes can promote wound healing by regulating cellular metabolic pathways. For example, plant-derived exosomes from ginseng significantly improve diabetic wound healing by enhancing glycolysis, reducing oxidative stress, and promoting angiogenesis in endothelial cells (Tan et al., 2024). Similarly, exosomes derived from mesenchymal stromal cells (MSCs) restore cellular bioenergetics and oxidative balance following ischemic injury, underscoring their therapeutic potential in metabolic modulation during tissue repair (Faria et al., 2023).

Saliva-derived exosomes (S-Exo), characterized by their abundant bioactive constituents and accessibility, have also shown promising therapeutic effects in wound healing. Prior studies have demonstrated that S-Exo accelerate skin wound closure by promoting angiogenesis, modulating inflammatory responses, and inducing favorable macrophage polarization (Mi et al., 2020; Qian et al., 2024). Although the potential of saliva-derived exosomes to enhance wound healing has been demonstrated, the detailed mechanisms by which they exert their effects remain largely unexplored. This study aims to investigate the specific role of S-Exo in modulating fibroblast metabolism, particularly focusing on glycolysis, and their subsequent impacts on fibroblast functionality, ECM remodeling, and MMP activity during wound healing. By integrating *in vitro* experiments and animal models, this research seeks to elucidate novel metabolic regulatory mechanisms of S-Exo and establish a scientific basis for developing innovative exosome-based therapies to enhance skin wound healing outcomes.

## Methods

### Animal model establishment

Healthy SPF Sprague-Dawley (SD) rats (8–10 weeks old, both sexes, *n* = 12, weighing 200–300 g) were anesthetized with 1%–3% isoflurane. After dorsal hair removal and disinfection (75% ethanol), two symmetrical full-thickness skin defects (1 × 1 cm^2^) were created using a surgical scalpel. Wounds were initially covered with Tegaderm™ (3M, United States) for 48 h. From day 2, wounds were kept open. Saliva-derived exosomes (S-Exos, 50 μg/wound) or PBS were injected subcutaneously around wounds every 3 days from day 2, 4 times (harvested at day 14) or 5 times (harvested at day 35). Animal experiments were approved by Guangxi Medical University Animal Ethics Committee.

### Cell culture

HaCaT keratinocytes (China Typical Culture Collection, Wuhan University, China) and human skin fibroblasts (HSFs), isolated from skin samples obtained from volunteers (age 18–65, without systemic or infectious diseases) undergoing flap surgeries at the Affiliated Stomatological Hospital of Guangxi Medical University, were cultured in DMEM supplemented with 10% fetal bovine serum (FBS, Gibco, United States) at 37°C, 5% CO_2_. HSFs between passages 3–6 were used for experiments. The protocol was approved by the Ethical Committee of Guangxi Medical University College of Stomatology.

### Extraction and characterization of S-Exos

Saliva samples from healthy adult volunteers were collected (9:00–12:00 a.m.) with informed consent and ethical approval (Guangxi Medical University College of Stomatology). Samples diluted in PBS were sequentially centrifuged at 3,000×g (30 min), 10,000×g (40 min), filtered (0.22 μm, Millipore, United States), and ultracentrifuged twice at 120,000×g (90 min, 4°C). Exosomes were resuspended in PBS. Protein concentration was determined using a BCA assay kit (Thermo Fisher, United States). Exosome morphology and particle size distribution were characterized by transmission electron microscopy (TEM, hitachi, Japan) and nanoparticle tracking analysis (NanoSight, Particle Metrix, Germany).

### Wound healing assessment

Wounds were photographed at days 0, 3, 5, 7, 10, 11, and 14 post-surgery. Wound areas were quantified using ImageJ software, and the relative wound area (%) was calculated as (unhealed area/original area) × 100%.

### Histological analysis

Paraffin-embedded skin tissues were sectioned (4 μm) and subjected to Hematoxylin-eosin (H&E) staining for general histology and Sirius Red staining for collagen deposition. Immunohistochemistry (IHC) and immunofluorescence staining were conducted following standard protocols, including antigen retrieval, primary antibody incubation, HRP-conjugated secondary antibodies incubation, visualization with DAB (IHC), and TSA-fluorescent substrates and DAPI counterstaining (immunofluorescence). Images were captured using fluorescence microscopy.

### Transwell co-culture model

HaCaT cells and HSFs were co-cultured using 6-well Transwell inserts (0.4 μm pore size, Corning, United States). HSFs were seeded in the lower chamber and HaCaT cells in the upper insert. Cells were grouped: HaCaT alone, HSFs alone, and co-culture, with or without S-Exos (50 μg/mL). Cell lysates and supernatants were collected for Western blotting and ELISA.

### Cell functional assays

For proliferation assays, cells treated with S-Exos (50 μg/mL) or PBS were analyzed using CCK-8 (UElandy, China) at 24, 48, and 72 h. Scratch assays assessed cell migration at 0, 24, and 48 h, analyzed by ImageJ. Transwell migration assays used 8 μm pore inserts (Corning, United States), with cells fixed and stained for quantification after 24 h.

### ELISA

Secreted proteins were measured in culture supernatants using ELISA kits (Multi Sciences, China) per manufacturer’s protocol. Absorbance was measured at 450 nm.

### Western blotting

Proteins were extracted, quantified using BCA assay, separated by SDS-PAGE, transferred to PVDF membranes (Millipore, United States), incubated with primary antibodies, and visualized with chemiluminescence detection (ECL, Beyotime, United States). Antibody details are listed in Table 1.

**TABLE 1:** Antibodies used in this study.

Target protein	Supplier
CD9	System biosciences
CD63	System biosciences
HSP70	Servicebio
Calnexin	Abcam
Collagen I	Proteintech
Collagen III	Proteintech
α-SMA	Proteintech
MMP1	Abmart
MMP3	Servicebio
TIMP1	Zen-Bioscience
HK2	Servicebio
β-actin	Servicebio

### RT-qPCR

RNA was extracted (Trizol), cDNA synthesized, and PCR performed using SYBR Green qPCR reagents (Genstar, China). Expression levels were normalized to β-actin. Primer sequences are listed in Table 2.

**TABLE 2:** Primer sequences used for RT-qPCR.

Gene	Forward primer (5′–3′)	Reverse primer (5′–3′)
MMP1	AGGTCTCTGAGGGTCAAGCA	CTGGTTGAAAAGCATGAGCA
MMP3	CTGGGCCAGGGATTAATGGAG	GGCCAATTTCATGAGCAGCA
MMP13	TACCAGACTTCACGATGGCATTGCTG	AAAGTGGCTTTTGCCGGTGTAGGTG
MMP2	TACAGGATCATTGGCTACACACC	GGTCACATCGCTCCAGACT
TIMP1	AATTCCGACCTCGTCATCAG	TGCAGTTTTCCAGCAATGAG
HK2	GAGCCACCACTCACCCTACT	CCAGGCATTCGGCAATGTG
PKM2	ATTATTTGAGGAACTCCGCCGCCT	ATTCCGGGTCACAGCAATGATGG
LDHA	ATCTTGACCTACGTGGCTTGGA	CCATACAGGCACACTGGAATCTC
PDK1	ACCAGGACAGCCAATACAAG	CCTCGGTCACTCATCTTCAC
UCP3	CATCATGAGGAATGCTATCG	TGGAGGTGAGTTCATATACC
GLUT1	ATTGGCTCCGGTATCGTCAAC	GCTCAGATAGGACATCCAGGGTA
PFKM	GACCCGTGGTTCTCGTCTC	AAAGGCTGATGGCGTCCC
NDUFS1	TTAGCAAATCACCCATTGGACTG	CCCCTCTAAAAATCGGCTCCTA
COX5A	GTCACAGGAGACAGATGAGGAGTTT	AAAGTTGGTCTAAGTTCCTGGATGA
SOD2	CCCAGATAGCTCTTCAGCCTGCACT	TAAGCGTGCTCCCACACATCAATCC
TIMP2	AAGCGGTCAGTGAGAAGGAAG	GGGGCCGTGTAGATAAACTCTAT
β-actin	TGGCACCCAGCACAATGAA	CTAAGTCATAGTCCGCCTAGAAGCA

### Experimental design for metabolic pathway analysis of HSFs

To explore the role of S-Exos in metabolic regulation of HSFs, HSFs were treated with PBS, S-Exos (50 μg/mL), insulin (2 μg/mL), 2-DG (2 mM), NMN (100 μM), or oligomycin (5 nM) alone or combined with S-Exos.

### RNA sequencing and bioinformatics analysis

RNA sequencing was performed by LC-Bio Technology Co., Ltd. (Hangzhou, China). Briefly, total RNA extracted (Trizol) from tissues underwent quality assessment (Agilent 2100, RIN >7.0), library preparation, and sequencing (Illumina Novaseq™ 6000, paired-end 2 × 150 bp). Differential gene expression analysis was conducted using DESeq2 (FDR <0.05, |log2FC|≥1). Gene Set Enrichment Analysis (GSEA) and visualizations were performed using R software (v4.2.0).

### ATP measurement

HSFs were treated with different media for 12 h. Cells were then lysed, and ATP was quantified using an ATP assay kit (Beyotime, China), Luminescence signals were measured with a multimode reader and normalized to total protein (nmol ATP/mg protein).

### Glucose measurement

Cell culture supernatants were collected and glucose concentrations were determined using a glucose assay kit (Nanjing Jiancheng, China). Absorbance was measured at 505 nm. Glucose consumption was calculated by subtracting residual levels from the initial medium concentration.

### LC-MS targeted metabolomics

Metabolite extraction and targeted LC-MS analysis (Waters BEH Amide column, Waters) were performed by LC-Bio Technology Co., Ltd. (Hangzhou, China). Mass spectrometry operated in positive and negative ion modes. Internal standards and QC samples were used for data normalization.

### DIA proteomics and bioinformatics analysis

DIA proteomic analysis was conducted by Shanghai Applied Protein Technology Co., Ltd. (Shanghai, China). Proteins underwent SDS-PAGE, digestion (FASP method), peptide desalting, and analysis by Orbitrap Astral™ mass spectrometer (Thermo Fisher Scientific, United States) coupled with Vanquish Neo nano-LC. Bioinformatics analysis and visualizations were performed using R software (version 4.2.2).

### Statistical analysis

Statistical analyses were performed using GraphPad Prism (version 10.0). Data were expressed as mean ± SD. Differences were evaluated using t-tests (two groups) or ANOVA tests (multiple groups). P < 0.05 indicated statistical significance.

## Results

### S‐Exo accelerates wound healing

**FIGURE 1:**
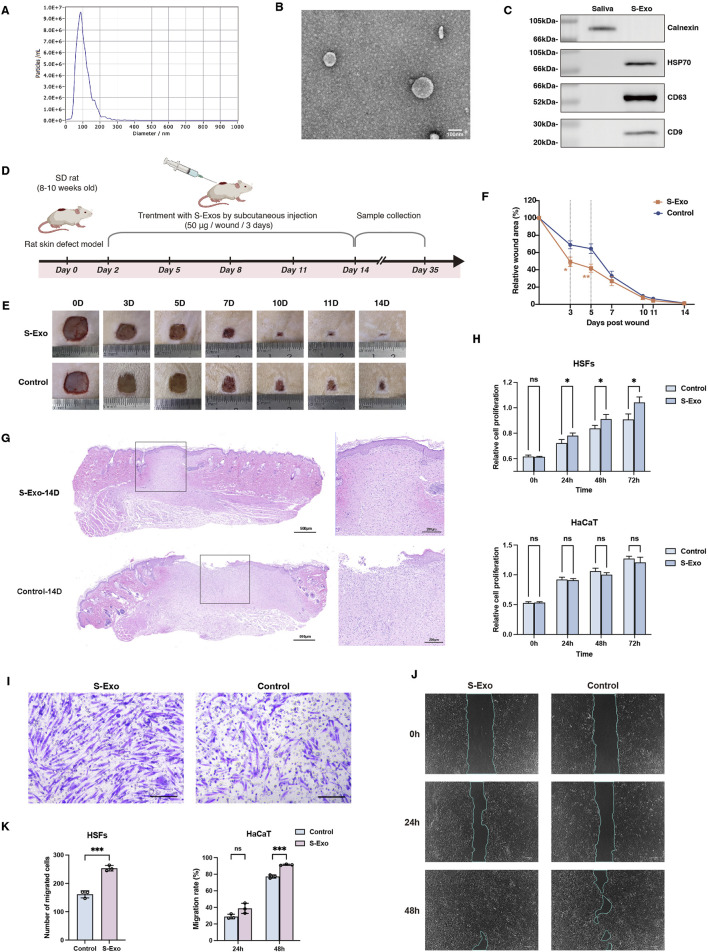
Characterization and impact of S-Exo on wound healing and cellular function. **(A)** NTA showing size distribution of S-Exo, with peak diameter around 100 nm. **(B)** TEM images of S-Exo displaying characteristic cup-shaped, membrane-bound vesicles (Scale bar = 100 nm). **(C)** Western blot analysis identifying exosomal markers CD9, CD63, HSP70 in S-Exo, and confirming absence of the endoplasmic reticulum marker calnexin. **(D)** Schematic of the *in vivo* wound healing assay with subcutaneous S-Exo injections. **(E)** Sequential photographs depicting wound healing progress at various time points post-treatment. **(F)** Wound closure rates over time (*n* = 12). **(G)** Histological sections of the wound at day 14 post-surgery stained with H&E. **(H)** CCK-8 assays showing proliferation of HaCaT cells and HSFs after S-Exo treatment (*n* = 5). **(I)** Transwell assay showing enhanced migration of HSFs with S-Exo treatment (Scale bar = 100 µm). **(J)** Scratch assay images illustrating improved HaCaT cell migration following S-Exo treatment. **(K)** Statistical analysis of migration capabilities in HaCaT cells and HSFs after S-Exo treatment (*n* = 3). (*P < 0.05, **P < 0.01, ***P < 0.001; ns indicates no significant difference).

S‐Exo were successfully isolated from saliva and rigorously characterized. NTA revealed that the S‐Exo particles predominantly ranged from 50 to 150 nm, with an average diameter of approximately 100 nm (Figure 1A). TEM confirmed their typical round, membrane-bound morphology (Figure 1B). Moreover, Western blot analysis demonstrated the specific enrichment of exosomal markers CD9, CD63, and HSP70 in S‐Exo, while the endoplasmic reticulum marker calnexin was undetectable (Figure 1C). Collectively, these findings validate that the isolated vesicles exhibit the expected biophysical and biochemical characteristics of exosomes.

To assess the therapeutic potential of S‐Exo in skin wound repair, both *in vivo* and *in vitro* experiments were performed. In a rat full‐thickness skin defect model, S‐Exo were administered via subcutaneous injections around the wound starting on post‐operative day 2 and repeated every 3 days for a total of four injections (up to day 11) (Figure 1D). Quantitative analysis revealed that S-Exo treatment significantly accelerated wound closure. Notably, wounds treated with S‐Exo showed a 50% reduction in wound area by day 3, whereas control wounds reached a similar reduction only after day 5 (Figures 1E,F). Histological analysis at day 14 post-surgery further revealed complete re‐epithelialization with markedly reduced inflammatory cell infiltration in the S‐Exo‐treated wounds, indicating an accelerated and more mature healing process (Figure 1G).

In parallel, *in vitro* studies using HSFs and HaCaT keratinocytes were conducted. Culturing these cells in medium supplemented with S‐Exo significantly enhanced HSF proliferation and migration ability (Figures 1H,I), and similarly improved the migratory capacity of HaCaT cells (Figures 1J,K). Taken together, these results suggest that S‐Exo promote wound healing by enhancing the functions of both fibroblasts and keratinocytes, thereby facilitating rapid tissue repair.

### S-Exo enhances ECM remodeling by promoting MMP secretion

**FIGURE 2:**
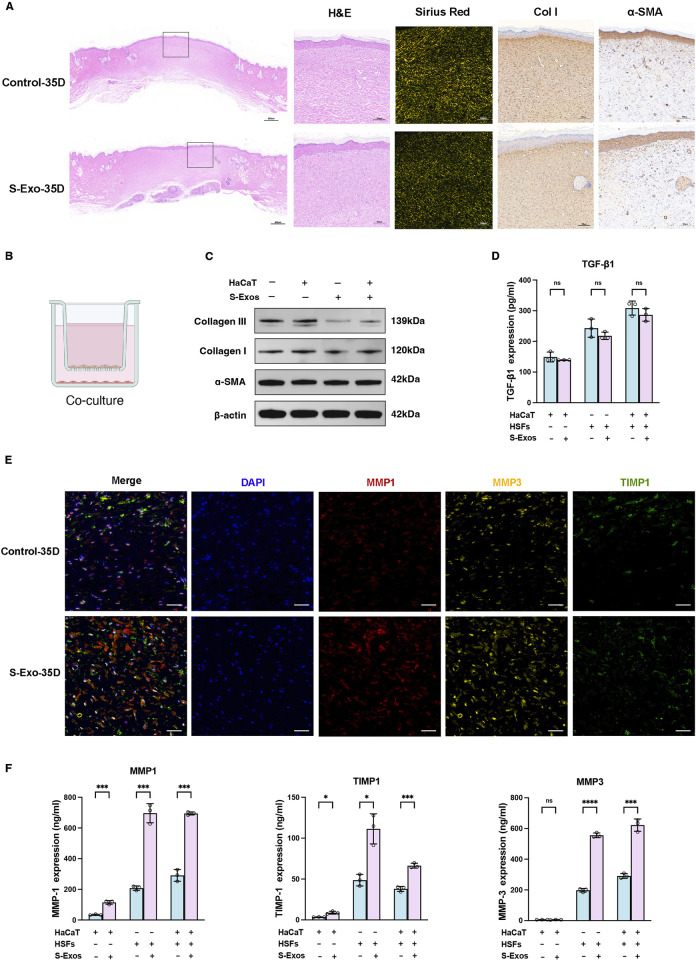
Impact of S-Exo on ECM remodeling and MMPs regulation in skin wound healing. **(A)** Histological comparisons of control and S-Exo-treated wounds at 35 days, stained with H&E, Sirius Red, Collagen I, and α-SMA. **(B)** Diagram of the 3D co-culture system for HaCaT and HSFs with S-Exo treatment. **(C)** Western blot analysis of fibrosis markers Collagen I, Collagen III, and α-SMA in HSFs co-cultured with S-Exo. **(D)** TGF-β1 levels in co-culture supernatants measured by ELISA (*n* = 3). **(E)** Immunofluorescence staining for MMP1, MMP3, and TIMP1 in skin sections at 35 days post-treatment (scale bar = 100 µm). **(F)** MMP1, MMP3, and TIMP1 expression in co-culture systems measured by ELISA (*n* = 3). (*P < 0.05, **P < 0.01, ***P < 0.001, ****P < 0.0001; ns indicates no significant difference).

To investigate the long-term impact of S‐Exo on wound healing and scar formation, wound tissues were harvested at post-operative day 35. H&E staining revealed that, compared with controls, S‐Exo-treated wounds exhibited collagen fibers that were finer and more evenly distributed, whereas control wounds displayed thick, bundled collagen fibers (Figure 2A). Consistently, collagen I IHC staining confirmed a more uniform collagen architecture in the S‐Exo group, and there was no significant alteration in α-SMA expression, indicating that myofibroblast activation was not enhanced. Moreover, Sirius Red staining under polarized light demonstrated that collagen fibers in control wounds displayed an orange-yellow spectrum—indicative of thicker, highly cross-linked fibers—whereas S‐Exo-treated wounds showed a yellow-green spectrum corresponding to thinner, less cross-linked collagen. Occasional regeneration of sebaceous glands was also observed in the S‐Exo group, suggesting a potential restoration of skin appendages. These findings collectively indicate that S‐Exo improves collagen organization and may reduce abnormal scar formation.

To further explore the effects of S‐Exo on ECM synthesis and remodeling, a Transwell co-culture system was established using HaCaT keratinocytes and HSFs to mimic epithelial–dermal interactions during wound repair (Figure 2B). Western blot analysis revealed that S‐Exo treatment modestly reduced the expression of collagen I and collagen III in HSFs, while leaving α-SMA levels unchanged (Figure 2C). ELISA assays confirmed that the secretion of TGF-β1 was not significantly altered, suggesting that the anti-scarring effects of S‐Exo may operate independently of the TGF-β/SMAD pathway (Figure 2D).

Given the critical role of MMPs in ECM remodeling, we then focused on the expression of MMP1, MMP3, and their inhibitor TIMP1. Immunofluorescence staining on day 35 showed that S‐Exo treatment markedly enhanced the expression of MMP1 and MMP3, with a concurrent moderate increase in TIMP1 (Figure 2E). Quantitative ELISA analysis revealed that, compared with controls, HSFs in the S‐Exo-treated group secreted MMP1 and MMP3 at levels 3.35-fold (*P* < 0.001) and 2.81-fold (*P* < 0.0001) higher, respectively, while TIMP1 levels increased by 2.28-fold (*P* < 0.05) (Figure 2F). RT-qPCR further confirmed that multiple MMP family members were upregulated in S‐Exo-treated HSFs. Among the two TIMP family members tested (TIMP1 and TIMP2), only TIMP1 exhibited a significant increase in expression, resulting in an overall elevated MMP/TIMP ratio (Supplementary Figure S1). This shift toward enhanced ECM degradation suggests that S‐Exo promote dynamic ECM remodeling, thereby preventing abnormal ECM accumulation and subsequent scar formation.

### S-Exo restores metabolic pathways and enhances glycolysis at the wound site

**FIGURE 3:**
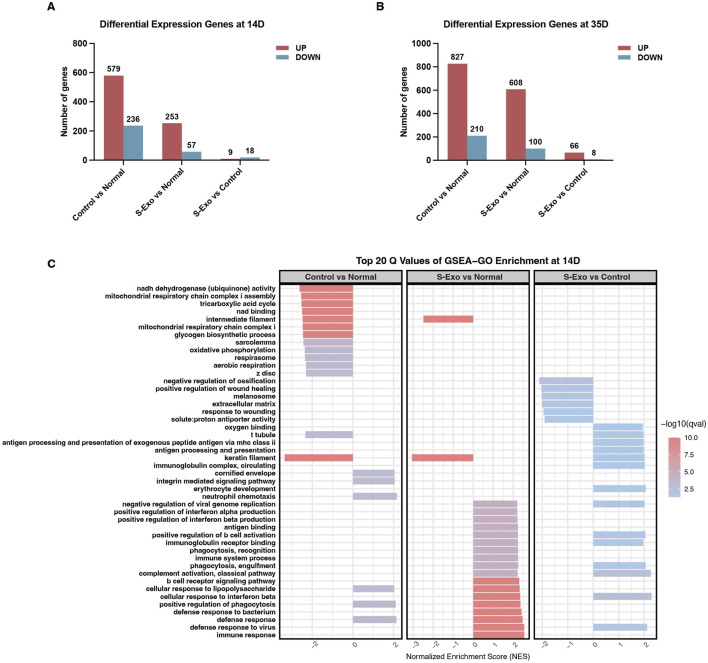
Gene expression and GSEA-GO enrichment analysis in S-Exo-treated wounds. **(A,B)** Differential gene expression at 14 and 35 days, comparing S-Exo-treated, Control, and Normal skin samples. **(C)** Top 20 GSEA-GO enrichment terms at day 14 for the indicated comparisons.

To investigate the transcriptomic changes associated with S‐Exo treatment during wound healing, wound tissues were collected at post-operative days 14 and 35 from both S‐Exo-treated and control groups, with normal skin serving as the reference. Differential expression analysis revealed that the S‐Exo-treated wounds exhibited fewer differentially expressed genes (DEGs) compared to normal skin than did control wounds. Specifically, 310 DEGs were identified in the S‐Exo group versus 815 in the control group at day 14, and 708 versus 1037 DEGs at day 35 (Figures 3A,B). This reduction in DEGs suggests that S‐Exo treatment restores the gene expression profile of wounded tissue to a state closer to that of normal skin, thereby improving its molecular environment.

Subsequent GSEA using Gene Ontology (GO) terms on day 14 samples revealed that S‐Exo-treated wounds exhibited significant enrichment in immune-related processes, such as defense responses and antigen processing, when compared with normal skin (Figure 3C). Notably, control wounds demonstrated a marked downregulation of energy metabolism functions, particularly those associated with mitochondrial respiration and the tricarboxylic acid (TCA) cycle. However, this pattern was not observed in the S‐Exo group; on the contrary, S‐Exo-treated samples showed a trend toward enhanced oxygen-binding capacity. Furthermore, biological processes related to ECM organization and cell migration, including integrin-mediated signaling, were more pronounced in the S‐Exo group. Collectively, these results suggest that S‐Exo treatment modulates immune responses, preserves energy metabolism, and influences ECM remodeling, thus contributing to improved wound healing quality.

**FIGURE 4:**
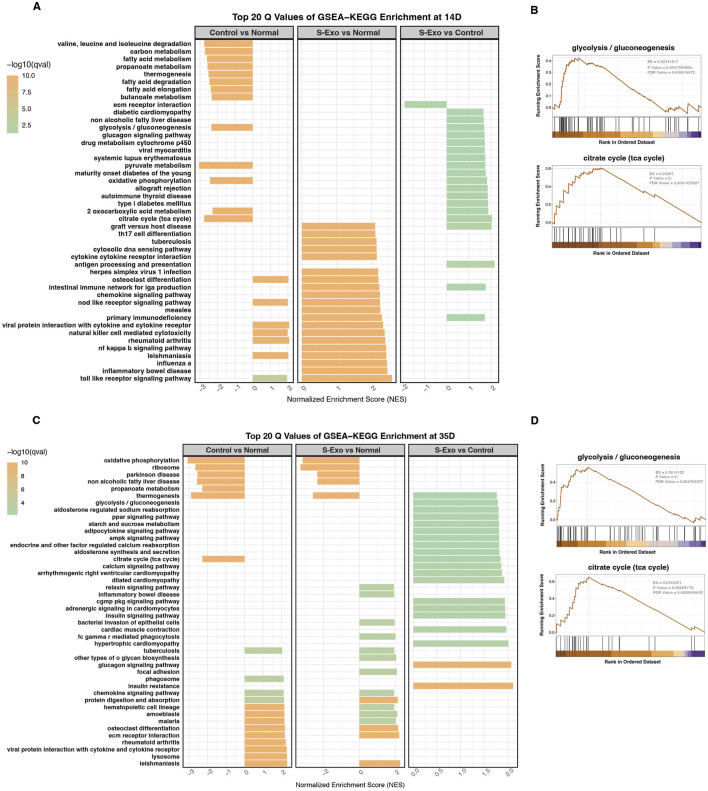
Pathway analysis through GSEA-KEGG enrichment at 14 and 35 days. **(A,C)** GSEA-KEGG enrichment analysis at 14 days **(A)** and 35 days **(C)** showing top 20 enriched pathways for Control vs. Normal, S-Exo vs. Normal, and S-Exo vs. Control. **(B,D)** GSEA plots for glycolysis/gluconeogenesis and the citrate cycle (TCA cycle) at 14 days **(B)** and 35 days **(D)** for S-Exo vs. Control.

KEGG pathway analysis using GSEA further underscored these findings. At day 14, control wound tissues exhibited systematic downregulation of key energy metabolism pathways when compared with normal skin (Figure 4A). In contrast, the S‐Exo-treated wounds maintained these pathways at levels comparable to normal skin. Moreover, direct comparisons between the S‐Exo and control groups revealed that S‐Exo treatment significantly activated glycolysis/gluconeogenesis (NES = 1.686, FDR = 0.039) and the TCA cycle (NES = 2.045, FDR = 0.006) (Figures 4A,B). At day 35, the S‐Exo group continued to show sustained upregulation of glycolysis/gluconeogenesis (NES = 1.788, FDR = 0.004) and the TCA cycle (NES = 1.847, FDR = 0.002), along with activation of pathways related to tissue remodeling, such as protein digestion and absorption and ECM-receptor interaction (Figures 4C,D). These findings suggest that S‐Exo treatment not only restores energy metabolism in wounded tissues but also supports subsequent tissue repair and ECM remodeling through the activation of critical glycolytic and metabolic pathways.

**FIGURE 5:**
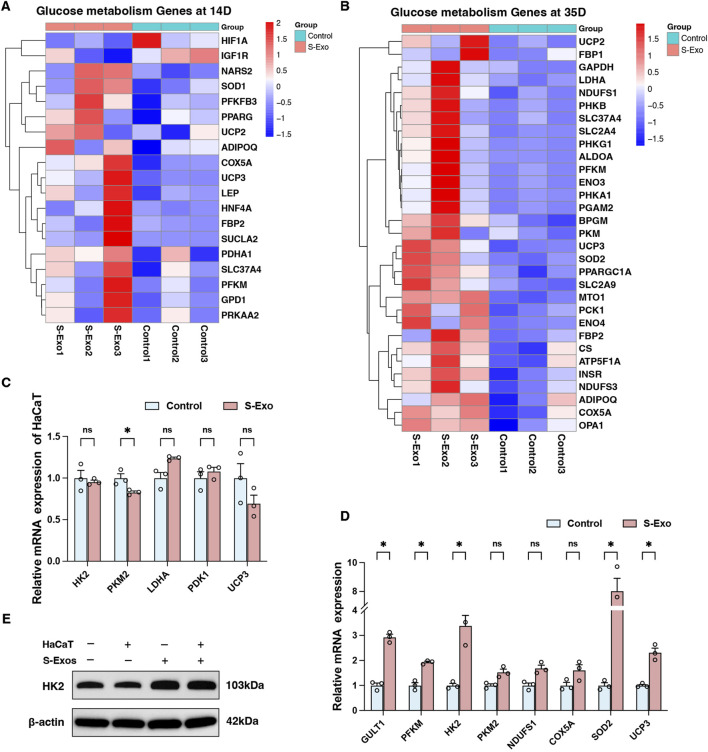
Transcriptional profiling of glucose metabolism genes and their regulation by S-Exo in HaCaT cells and HSFs. **(A,B)** Heatmaps illustrating transcriptional profiling of glucose metabolism genes at 14 days **(A)** and 35 days **(B)** post-S-Exo treatment. **(C,D)** RT-qPCR analysis showing the relative mRNA expression of selected glycolysis-related genes in HaCaT cells **(C)** and HSFs **(D)** (*n* = 3) after S-Exo treatment. **(E)** Western blot for HK2 protein expression in HSFs under co-culture conditions with or without S-Exo. (*P < 0.05; ns indicates no significant difference).

Detailed transcriptomic analysis revealed that at day 14 post-injury revealed that wound tissues from the S‐Exo-treated group exhibited significant upregulation of key glycolytic enzyme genes such as PFKM and PFKFB3 (Figure 5A). In addition, genes related to the TCA cycle and mitochondrial function (e.g., SUCLA2, PDHA1, and NARS2), glucose mobilization (FBP2 and GPD1), and antioxidant defense (SOD1, UCP2, and UCP3) were also upregulated. This coordinated response likely meets the increased energy and biosynthetic demands during early wound repair.

At day 35, S‐Exo-treated wounds continued to display high expression of glycolytic genes (e.g., GAPDH, PFKM, LDHA, and ENO3), mitochondrial respiratory chain genes (e.g., CS, NDUFS1, NDUFS3, COX5A, and ATP5F1A), glucose mobilization genes (e.g., FBP1, FBP2, PCK1, and G6PC), as well as antioxidant genes (SOD1, UCP2, and UCP3) (Figure 5B). These findings emphasize the sustained activation of energy metabolism in the wound environment following S‐Exo treatment.

To further validate these transcriptomic results, an *in vitro* model using HaCaT cells and HSFs was employed. RT-qPCR analysis indicated that S‐Exo treatment did not significantly alter the expression of glucose metabolism-related genes in HaCaT cells (Figure 5C). In contrast, HSFs exhibited significant upregulation of key glycolytic genes, including GLUT1, PFKM, and HK2, with HK2 mRNA levels increasing 3.385-fold compared with controls (*P* < 0.05) (Figure 5D). Western blot analysis confirmed the increased expression of HK2 protein in HSFs after S‐Exo treatment (Figure 5E). Furthermore, HSFs also showed an upward trend in the expression of mitochondrial respiratory chain genes (NDUFS1 and COX5A) and antioxidant genes (SOD2 and UCP3). These results suggest that S‐Exo specifically reprogram fibroblast metabolism, providing sustained energy and biosynthetic support that likely contributes to effective wound healing.

### S-Exo induces glycolytic shift to regulate fibroblast metabolism and MMP secretion

**FIGURE 6:**
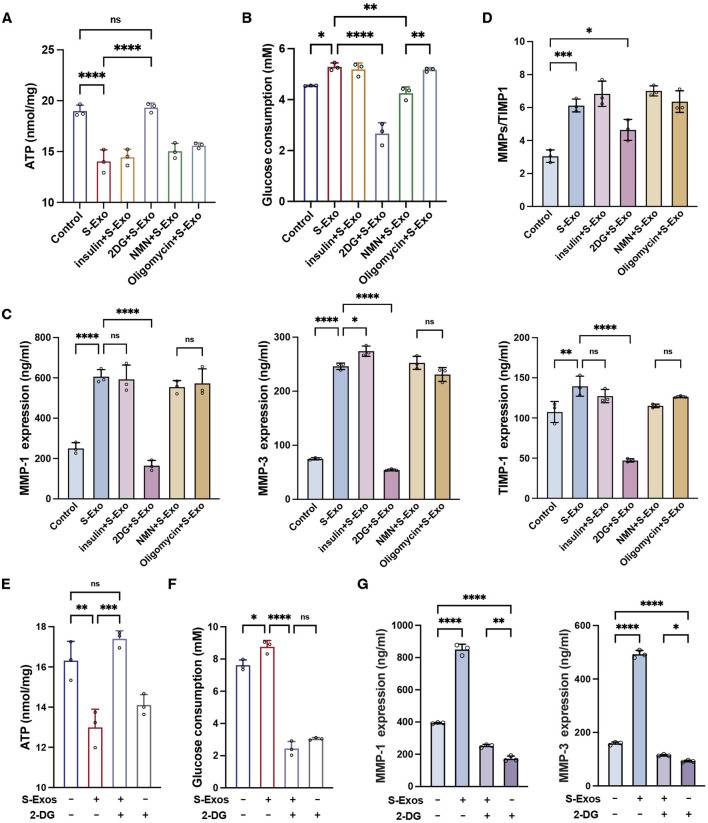
S-Exo shifts HSF energy metabolism to glycolysis and enhances MMP secretion. **(A)** ATP levels in cells treated with S-Exo alone or in combination with insulin, 2-DG, NMN, and oligomycin. **(B)** Glucose consumption in culture media from HSFs treated with S-Exo and metabolic modulators. **(C)** Expression levels of MMP-1, MMP-3, and TIMP1 in culture media from HSFs treated with S-Exo and metabolic modulators. **(D)** Ratio of MMP-1 and MMP-3 to TIMP1. **(E)** Effects of S-Exo treatment on ATP levels with and without the presence of 2-DG. **(F)** Effects of S-Exo treatment on glucose consumption in HSFs with and without 2-DG. **(G)** Comparative expression of MMP-1 and MMP-3 in culture media from HSFs treated with S-Exo and 2-DG. (*P < 0.05, **P < 0.01, ***P < 0.001, ****P < 0.0001; ns indicates no significant difference).

To investigate the role of glycolytic flux in mediating the effects of S‐Exo on fibroblast energy metabolism and MMP secretion, HSFs were treated with S‐Exo in combination with various metabolic modulators, including insulin (a glycolysis activator), 2‐deoxyglucose (2‐DG, a glycolysis inhibitor), nicotinamide mononucleotide (NMN, a mitochondrial respiration activator), and oligomycin (a mitochondrial respiration inhibitor). S‐Exo treatment significantly increased glucose consumption by HSFs while concurrently reducing intracellular ATP levels (Figures 6A,B), suggesting that S‐Exo shifted cellular energy production toward glycolysis. Co-treatment with 2‐DG decreased glucose consumption by 49.6% (*P* < 0.0001) and increased ATP levels 1.38-fold (*P* < 0.0001) compared with S‐Exo alone, whereas treatments with insulin, NMN, or oligomycin did not produce significant differences relative to S‐Exo treatment.

When S‐Exo-treated HSFs were co-treated with 2‐DG, the secretion of MMP1, MMP3, and TIMP1 was significantly reduced by 72.8%, 77.9%, and 66.1% (*P* < 0.0001) relative to S‐Exo treatment alone (Figure 6C). Moreover, while S‐Exo alone increased the (MMP1 + MMP3)/TIMP1 ratio by 2.0-fold (*P* < 0.001), the addition of 2‐DG decreased this ratio to 1.5-fold (*P* < 0.05) (Figure 6D). These results indicate that the inhibition of glycolysis with 2‐DG significantly attenuated the S‐Exo-induced enhancement of MMP secretion, underscoring the critical role of glycolytic flux in regulating ECM degradation.

Additionally, treatment of HSFs with 2‐DG alone resulted in a significant decrease in intracellular ATP (*P* < 0.05) and a reduction in glucose consumption of approximately 60.1% (*P* < 0.0001) (Figures 6E,F), confirming the effective inhibition of glycolysis by 2‐DG. In contrast, co-treatment with S‐Exo and 2‐DG increased ATP levels by approximately 1.23-fold compared with 2‐DG alone (*P* < 0.01) (Figure 6G). Moreover, under the combined treatment condition, the inhibitory effects of 2‐DG on MMP1 and MMP3 secretion were partially reversed, with reversal percentages of 35.9% and 33.0%, respectively (Figure 6G). Thus, these data suggest that although S‐Exo can activate compensatory metabolic pathways to partially restore energy supply under glycolytic inhibition, glycolytic flux remains the primary mechanism driving MMP production.

**FIGURE 7:**
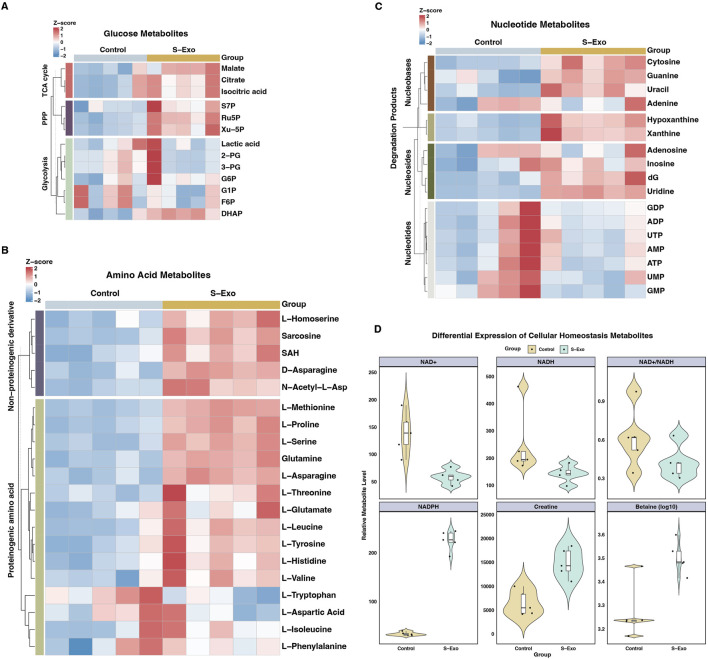
Metabolic profiling of S-Exo effects on HSFs across key metabolic pathways. **(A)** Heatmaps displaying concentrations of glucose metabolism-related metabolites in HSFs treated with S-Exo. **(B)** Heatmaps showing the concentrations of amino acid metabolism-related metabolites in HSFs under the influence of S-Exo. **(C)** Heatmaps detailing the concentration changes in nucleotide metabolism-related metabolites in HSFs treated with S-Exo versus control. **(D)** Violin plots illustrating the levels of redox metabolites NAD+, NADH, NADPH, Creatine and Betaine in S-Exo-treated versus control HSFs (*n* = 5).

To further validate whether S‐Exo treatment induces extensive metabolic reprogramming in HSFs and to elucidate its effects on biosynthetic pathways, we performed LC–MS targeted metabolomics analysis, comparing control and S‐Exo-treated groups. The analysis revealed significant changes in key metabolic pathways, indicating a global reprogramming of cellular metabolism (Figures 7A–D; Tables 3–6).

**TABLE 3:** Relative levels of glucose metabolites in HSFs after S-Exo treatment.

Metabolite class	Metabolite	Control (x̅ ± s)	S-Exo (x̅ ± s)	S-Exo vs. Control
Glycolysis/gluconeogenesis	Glucose-1-phosphate (G1P)	26.6 ± 3.0	24.4 ± 2.3	0.92-fold (ns)
Glycolysis/gluconeogenesis	Fructose-6-phosphate (F6P)	28.4 ± 3.3	26.6 ± 2.1	0.94-fold (ns)
Glycolysis/gluconeogenesis	Glucose-6-phosphate (G6P)	152 ± 28.6	187 ± 35.8	1.23-fold (ns)
Glycolysis/gluconeogenesis	Dihydroxyacetone phosphate (DHAP)	16.4 ± 3.5	24.6 ± 1.7	1.50-fold (**)
Glycolysis/gluconeogenesis	3-Phosphoglycerate (3-PG)	1292.2 ± 307.6	1255.4 ± 950.3	0.97-fold (ns)
Glycolysis/gluconeogenesis	2-Phosphoglycerate (2-PG)	1208.8 ± 287.2	1187.4 ± 922.5	0.98-fold (ns)
Glycolysis/gluconeogenesis	Lactic acid	436.4 ± 135.1	438.4 ± 154.5	1.00-fold (ns)
TCA cycle	Citrate	5524 ± 1527.9	7647.6 ± 1091.7	1.38-fold (*)
TCA cycle	Isocitrate	6495 ± 1701.4	8885.6 ± 1195.9	1.37-fold (*)
TCA cycle	Malate	213.2 ± 35.5	288.2 ± 34.3	1.35-fold (**)
Pentose phosphate pathway	Sedoheptulose-7-phosphate (S7P)	38.2 ± 4.0	46.4 ± 5.3	1.21-fold (*)
Pentose phosphate pathway	Ribulose-5-phosphate (Ru5P)	70.4 ± 13.2	139.2 ± 20.4	1.98-fold (***)
Pentose phosphate pathway	Xylulose-5-phosphate (Xu-5P)	83.8 ± 11.2	152 ± 31.5	1.81-fold (**)

*Data are expressed as mean ± SD (*n* = 5). Statistical significance versus the control group is indicated as follows: *P < 0.05, **P < 0.01, ***P < 0.001; “ns” denotes no significant difference.

**TABLE 4:** Relative levels of amino acid metabolites in HSFs after S-Exo treatment.

Metabolite class	Metabolite	Control (x̅ ± s)	S-Exo (x̅ ± s)	S-Exo vs. Control
Proteinogenic amino acids	L-Serine	127.6 ± 22.9	287.8 ± 21.5	2.26-fold (****)
Proteinogenic amino acids	L-Asparagine	19.4 ± 2.3	31.8 ± 1.5	1.64-fold (****)
Proteinogenic amino acids	L-Methionine	340.8 ± 34.3	686.8 ± 30.1	2.02-fold (****)
Proteinogenic amino acids	L-Proline	2612 ± 376.7	5027.2 ± 396.3	1.92-fold (****)
Proteinogenic amino acids	L-Threonine	59.6 ± 4.7	83.4 ± 13.5	1.40-fold (**)
Proteinogenic amino acids	L-Glutamate	44.8 ± 15.6	73.6 ± 21.4	1.64-fold (*)
Branched-chain amino acids	L-Valine	647.6 ± 69.3	811.6 ± 76.3	1.25-fold (**)
Branched-chain amino acids	L-Leucine	1695.6 ± 222.4	2359.2 ± 220.2	1.39-fold (**)
Proteinogenic amino acids	L-Histidine	223.4 ± 28.1	297.2 ± 35.3	1.33-fold (**)
Proteinogenic amino acids	L-Tyrosine	485.2 ± 67.1	630.0 ± 63.3	1.30-fold (**)
Proteinogenic amino acids	L-Tryptophan	256.8 ± 42.4	185.0 ± 37.3	0.72-fold (*)
Proteinogenic amino acids	L-Aspartic Acid	142.4 ± 18.9	133.8 ± 21.0	0.94-fold (ns)
Proteinogenic amino acids	L-Phenylalanine	2519.6 ± 277.7	2577.0 ± 64.8	1.02-fold (ns)
Branched-chain amino acids	L-Isoleucine	2044.2 ± 473.1	2360.4 ± 284.7	1.15-fold (ns)
Proteinogenic amino acids	Glutamine	166.2 ± 29.4	343.6 ± 23.2	2.07-fold (****)
Non-proteinogenic/modified derivatives	N-Acetyl-L-aspartate	72.8 ± 5.6	105.6 ± 10.0	1.45-fold (***)
Non-proteinogenic/modified derivatives	D-Asparagine	20.4 ± 2.8	33.2 ± 1.3	1.63-fold (****)
Non-proteinogenic/modified derivatives	Sarcosine	51.0 ± 7.5	101.2 ± 10.2	1.98-fold (****)
Non-proteinogenic/modified derivatives	S-Adenosylhomocysteine (SAH)	51.8 ± 10.6	82.0 ± 9.2	1.58-fold (**)
Non-proteinogenic/modified derivatives	L-Homoserine	96.2 ± 8.7	124.4 ± 8.9	1.29-fold (***)

*Data are expressed as mean ± SD (*n* = 5). Statistical significance versus the control group is indicated as follows: *P < 0.05, **P < 0.01, ***P < 0.001, ****P < 0.0001; “ns” denotes no significant difference.

**TABLE 5:** Relative levels of nucleotide metabolites in HSFs after S-Exo treatment.

Metabolite class	Metabolite	Control (x̅ ± s)	S-Exo (x̅ ± s)	S-Exo vs. Control
Nucleobase	Cytosine	19.0 ± 0.7	24.8 ± 1.8	1.31-fold (***)
Nucleobase	Guanine	2202.4 ± 1171.4	4330.6 ± 387.7	1.97-fold (**)
Nucleobase	Adenine	1713.0 ± 594.5	1807.8 ± 454.2	1.06-fold (ns)
Nucleobase	Uracil	41.2 ± 9.5	82.8 ± 14.4	2.01-fold (***)
Nucleoside	dG	22.4 ± 2.2	32.6 ± 3.4	1.46-fold (***)
Nucleoside	Uridine	186.8 ± 28.2	635.4 ± 32.4	3.40-fold (****)
Nucleoside	Adenosine	7493.2 ± 2722.9	8052.2 ± 2210.7	1.07-fold (ns)
Nucleoside	Inosine	28193.4 ± 10141.1	36065.6 ± 4697.4	1.28-fold (ns)
Nucleotide	GMP	109.4 ± 27.3	68.4 ± 6.9	0.63-fold (*)
Nucleotide	UMP	31.6 ± 8.5	26.0 ± 4.9	0.82-fold (ns)
Nucleotide	GDP	88.4 ± 44.8	74.8 ± 5.1	0.85-fold (ns)
Nucleotide	ADP	442.8 ± 216.2	384.6 ± 46.4	0.87-fold (ns)
Nucleotide	ATP	1041.6 ± 740.5	686.4 ± 338.9	0.66-fold (ns)
Nucleotide	AMP	43.6 ± 13.6	39.6 ± 6.0	0.91-fold (ns)
Nucleotide	UTP	189.4 ± 118.8	158.0 ± 58.3	0.83-fold (ns)
Degradation product	Xanthine	121.4 ± 12.5	254.6 ± 48.9	2.10-fold (***)
Degradation product	Hypoxanthine	426.6 ± 84.0	948.4 ± 159.8	2.22-fold (***)

*Data are expressed as mean ± SD (*n* = 5). Statistical significance versus the control group is indicated as follows: *P < 0.05, **P < 0.01, ***P < 0.001, ****P < 0.0001; “ns” denotes no significant difference.

**TABLE 6:** Relative levels of cellular homeostasis metabolites in HSFs after S-Exo treatment.

Metabolite class	Metabolite	Control (x̅ ± s)	S-Exo (x̅ ± s)	S-Exo vs. Control
Redox homeostasis	NAD^+^	139.2 ± 38.2	58.8 ± 12.8	0.42-fold (**)
Redox homeostasis	NADH	249.2 ± 121.0	143.6 ± 30.9	0.58-fold (ns)
Redox homeostasis	NAD^+^/NADH	0.6 ± 0.2	0.4 ± 0.1	0.69-fold (ns)
Redox homeostasis	NADPH	34.0 ± 3.7	225.6 ± 20.6	6.64-fold (****)
Redox homeostasis	GSSG	128.4 ± 38.9	140.8 ± 13.6	1.10-fold (ns)
Redox homeostasis	GSH	278.2 ± 83.0	326.4 ± 51.5	1.17-fold (ns)
Osmoregulation	Betaine	1911.8 ± 575.5	3207.0 ± 510.4	1.68-fold (**)
Energy buffer	Creatine	6469.6 ± 2577.0	14875.4 ± 3041.1	2.30-fold (**)
Redox homeostasis	5-Oxoproline	455.8 ± 208.7	536.8 ± 150.3	1.18-fold (ns)
Coenzyme metabolism	Biotin	74.8 ± 14.1	143.2 ± 12.0	1.91-fold (****)

*Data are expressed as mean ± SD (*n* = 5). Statistical significance versus the control group is indicated as follows: *P < 0.05, **P < 0.01, ***P < 0.001, ****P < 0.0001; “ns” denotes no significant difference.

S‐Exo treatment enhanced glycolytic and TCA cycle flux (Figure 7A; Table 3). For example, dihydroxyacetone phosphate (DHAP) increased by approximately 1.5-fold (*P* < 0.01), while key TCA cycle intermediates, including citrate, isocitrate, and malate, rose by roughly 1.35 to 1.38-fold (all *P* < 0.05). In addition, metabolites from the PPP were markedly upregulated; ribulose 5-phosphate (Ru5P) increased nearly 2-fold and xylulose 5-phosphate (Xu-5P) by about 1.8-fold, indicating enhanced anabolic activity.

S‐Exo-treated HSFs exhibited increased levels of most amino acids (Figure 7B; Table 4). For instance, L-serine increased by 2.26-fold (*P* < 0.0001), and branched-chain amino acids rose between 1.25-fold and 1.39-fold (*P* < 0.01), supporting enhanced protein synthesis. In nucleotide metabolism, nucleobases and nucleosides such as uracil and uridine were markedly elevated by approximately 2-fold and 3.4-fold, respectively, reflecting active nucleic acid synthesis. In contrast, certain nucleotide pools were reduced (e.g., GDP, UTP, and GMP), likely due to increased utilization in biosynthetic processes (Figure 7C; Table 5).

S‐Exo treatment resulted in a significant shift in cellular energy and redox status (Figure 7D; Table 6). NADPH levels were dramatically elevated by about 6.64-fold (*P* < 0.0001), whereas NAD^+^ and NADH levels decreased to approximately 0.42-fold (*P* < 0.01) and 0.58-fold (not significant) of control values, respectively, resulting in a lower NAD^+^/NADH ratio. Furthermore, energy-buffering compounds such as creatine increased by 2.30-fold (*P* < 0.01) and osmolytes like betaine rose by 1.68-fold (*P* < 0.01).

Taken together, S‐Exo treatment induces coordinated metabolic reprogramming in HSFs, characterized by enhanced carbohydrate metabolism, increased amino acid and nucleotide biosynthesis, and optimized redox and energy homeostasis. These metabolic adaptations likely contribute to the improved wound healing observed in S‐Exo-treated tissues.

### Proteomic profiling reveals metabolic regulatory potential of S-Exo

**FIGURE 8:**
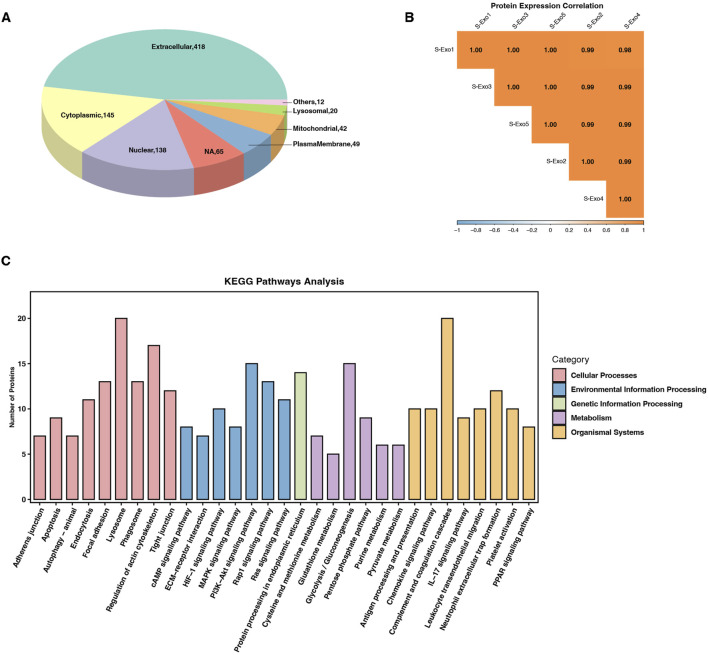
Proteomic profiling and pathway enrichment analysis of S-Exo. **(A)** Pie chart depicting the subcellular localization of proteins identified in S-Exo samples. **(B)** Heatmap illustrating the expression correlation among proteins from five S-Exo samples. **(C)** Bar chart showing KEGG pathway enrichment analysis of proteins identified in S-Exo.

To further investigate the molecular basis underlying S‐Exo-induced metabolic reprogramming in HSFs, we performed Oscar DIA proteomics on S‐Exo. A total of 751 proteins were identified from S‐Exo. Subcellular localization analysis revealed that these proteins were predominantly extracellular, with additional distribution in the cytoplasm, nucleus, mitochondria, and plasma membrane (Figure 8A). The protein expression correlations among samples ranged from 0.98 to 1.00, demonstrating high consistency and reproducibility (Figure 8B).

KEGG pathway enrichment analysis showed that proteins carried by S‐Exo were significantly enriched in core metabolic pathways including glycolysis/gluconeogenesis, the PPP, the TCA cycle, and amino acid metabolism. In addition, proteins related to platelet activation, complement and coagulation cascades, as well as signaling pathways involved in cell proliferation and migration (e.g., MAPK, PI3K-Akt, and Ras) were also significantly enriched. Pathways involved in ECM-receptor interactions and cell adhesion also showed notable enrichment (Figure 8C). These findings suggest that S‐Exo may regulate cellular functions through multiple signaling routes.

Focused analysis of proteins directly related to metabolic regulation revealed that S‐Exo was enriched in several key metabolic enzymes (Table 7). Notably, S‐Exo contained high levels of key glycolytic enzymes such as glyceraldehyde-3-phosphate dehydrogenase (GAPDH/GAPDHS), pyruvate kinase M2 (PKM), and enolase 1 (ENO1). Enzymes associated with the TCA cycle, including malate dehydrogenase (MDH1/2), and those playing critical roles in the PPP, including transketolase (TKT), 6-phosphogluconate dehydrogenase (PGD), and glucose-6-phosphate dehydrogenase (G6PD), were also significantly enriched. Furthermore, S‐Exo contained abundant enzymes involved in purine metabolism, such as purine nucleoside phosphorylase (PNP), nucleoside diphosphate kinase (NME2), and ecto-nucleoside pyrophosphatase/phosphodiesterase 3 (ENPP3), as well as proteins linked to glutathione metabolism, including glutathione S-transferase Pi (GSTP1) and peroxiredoxin 6 (PRDX6). These data indicate that S‐Exo may modulate the metabolic activity of recipient HSFs by delivering key enzymes involved in energy production and biosynthetic processes.

**TABLE 7:** Analysis of metabolic proteins in S-Exo by Oscar DIA proteomics.

KEGG pathway	Uniprot ID	Gene	Relative quantitation	Functional annotation
Glycolysis/gluconeogenesis	O14556	GAPDHS	2.02E+05	Glyceraldehyde-3-phosphate dehydrogenase; regulates glycolysis.
Glutathione metabolism	P09211	GSTP1	1.80E+05	GST Pi; catalyzes GSH conjugation for detoxification.
Glycolysis/gluconeogenesis	P06733	ENO1	1.57E+05	α-Enolase; converts 2-PG to PEP.
Glycolysis/gluconeogenesis; pyruvate metabolism; tyrosine metabolism	P08319	ADH4	1.41E+05	ADH4; reduces acetaldehyde to ethanol.
Glycolysis/gluconeogenesis	P60174	TPI1	1.07E+05	TPI1; interconverts DHAP and G3P.
Glycolysis/gluconeogenesis; cysteine and methionine; pyruvate metabolism	P00338	LDHA	7.21E+04	LDHA; converts pyruvate to lactate.
Purine metabolism	P22392	NME2	5.41E+04	NME2; synthesizes nucleoside triphosphates.
Glycolysis/gluconeogenesis; pentose phosphate pathway	P04075	ALDOA	5.25E+04	ALDOA; cleaves fructose-1,6-bisphosphate.
Tyrosine metabolism	P14174	MIF	5.12E+04	MIF; modulates inflammation.
Pentose phosphate pathway	P29401	TKT	4.93E+04	TKT; catalyzes sugar rearrangements in the PPP.
Glycolysis/gluconeogenesis; tyrosine metabolism	P30838	ALDH3A1	4.00E+04	ALDH3A1; oxidizes lipid aldehydes.
Cysteine and methionine; pyruvate metabolism	P40925	MDH1	3.99E+04	MDH1; converts malate and oxaloacetate.
Glycolysis/gluconeogenesis; pentose phosphate pathway	P06744	GPI	3.92E+04	GPI; interconverts G6P and F6P.
Alanine, aspartate and glutamate metabolism	P51649	ALDH5A1	3.79E+04	ALDH5A1; involved in GABA catabolism.
Glycolysis/gluconeogenesis	P04406	GAPDH	3.21E+04	GAPDH; key enzyme in glycolysis.
Cysteine and methionine; tyrosine; alanine, aspartate and glutamate metabolism	P17174	GOT1	3.16E+04	GOT1; mediates transamination reactions.
Glycolysis/gluconeogenesis	P00558	PGK1	2.51E+04	PGK1; produces ATP in glycolysis.
Glycolysis/gluconeogenesis; pyruvate metabolism	P14618	PKM	2.41E+04	PKM; converts PEP to pyruvate.
Alanine, aspartate and glutamate metabolism	P31327	CPS1	2.23E+04	CPS1; rate-limiting enzyme in the urea cycle.
Valine, leucine and isoleucine degradation	Q6NVY1	HIBCH	2.04E+04	HIBCH; degrades branched-chain amino acids.
Pentose phosphate pathway	P37837	TALDO1	1.97E+04	TALDO1; mediates sugar exchange in the PPP.
Glutathione metabolism	P30041	PRDX6	1.95E+04	PRDX6; reduces reactive oxygen species.
Cysteine and methionine; pyruvate metabolism	P40926	MDH2	1.88E+04	MDH2; oxidizes malate in mitochondria.
Purine metabolism	Q8WVQ1	CANT1	1.77E+04	CANT1; modulates purine nucleotide pools.
Pentose phosphate pathway; glutathione metabolism	P52209	PGD	1.55E+04	PGD; generates NADPH in the PPP.
Glutathione metabolism	P15144	ANPEP	1.38E+04	ANPEP; degrades glutathione peptides.
Pentose phosphate pathway; glutathione metabolism	P11413	G6PD	1.37E+04	G6PD; produces NADPH via the PPP.
Glycolysis/gluconeogenesis	P18669	PGAM1	1.17E+04	PGAM1; interconverts 2-PG and 3-PG.
Purine metabolism	P00491	PNP	9.65E+03	PNP; involved in purine salvage.
Glycolysis/gluconeogenesis; pentose phosphate pathway	P05062	ALDOB	8.54E+03	ALDOB; liver isoform regulating sugar metabolism.
Alanine, aspartate and glutamate metabolism	P15104	GLUL	6.90E+03	GLUL; synthesizes glutamine.
Cysteine and methionine; tyrosine; alanine, aspartate and glutamate metabolism	P00505	GOT2	5.80E+03	GOT2; involved in amino acid shuttling.
Purine metabolism	O14638	ENPP3	5.36E+03	ENPP3; hydrolyzes ATP to AMP.
Pentose phosphate pathway	O95479	H6PD	5.00E+03	H6PD; generates NADPH in the ER via the PPP.
Cysteine and methionine metabolism	Q99707	MTR	4.60E+03	MTR (methionine synthase); regenerates methionine.

## Discussion

Previous studies have demonstrated that fibroblasts and keratinocytes play critical roles during skin wound healing. Fibroblasts orchestrate ECM deposition and remodeling (Knoedler et al., 2023), while keratinocytes efficiently drive re-epithelialization, preventing infection and fluid loss (Rousselle et al., 2019). In this study, we found that S-Exo significantly enhanced early wound healing by activating fibroblasts and keratinocytes, thereby facilitating rapid tissue regeneration and epidermal barrier restoration (Figure 1). These findings suggest that S-Exo contains active molecules that promote cellular functions, which is consistent with observations in exosomes from other sources. For instance, exosomes derived from adipose-derived stem cells (ADSCs) similarly enhance fibroblast proliferation and keratinocyte migration, and promote angiogenesis at the wound site, thus accelerating wound closure in diabetic ulcers, likely due to the miRNA cargo they carry (Pomatto et al., 2021; Ma et al., 2019). Our proteomic analyses show that S-Exo contains active proteins, including metabolic enzymes, which may play a key role in promoting cell activity. Recent studies show that PKM2, a critical glycolytic enzyme, provides a metabolic advantage for regenerative cells, such as skin stem cells. It can activate keratinocytes in wounds, promoting proliferation, migration, and adhesion, thus accelerating re-epithelialization (Kim et al., 2023; Liu et al., 2025; Sych et al., 2023). However, the precise mechanisms through which these metabolic enzymes within S-Exo influence wound healing, particularly their effects on different cell types, remain to be fully elucidated. In particular, fibroblasts comprise multiple subtypes with distinct functions in wound healing, and whether S-Exo exert differential effects on these subpopulations remains an open and important question.

At later stages of wound healing, S-Exo treatment resulted in finer and more uniformly distributed collagen fibers, forming a flexible and well-organized matrix (Figure 2). This finding correlates with previous studies demonstrating enhanced MMP1 and MMP3 secretion, which selectively degrading thick type I collagen while preserving the more elastic type III collagen (Adhikari et al., 2012). Flexible matrices have been shown to feedback-regulate fibroblast homeostasis through mechanotransduction (Saraswathibhatla et al., 2023). Studies indicate that dermal fibroblasts cultured on soft substrates exhibit reduced myofibroblast activation and upregulated MMP1 and MMP3 expression, promoting ECM remodeling and tissue homeostasis (Achterberg et al., 2014). In such conditions, ibroblasts become more responsive to growth factors, preserving their regenerative capacity, and may activate Wnt/β-catenin signaling in epidermal stem cells, promoting sebaceous gland regeneration (Myung et al., 2022; Astudillo, 2020). Notably, these beneficial changes appeared independent of TGF‐β/SMAD signaling, as indicated by unchanged TGF‐β1 and α‐SMA levels, suggesting the involvement of alternative regulatory mechanisms. The elevated MMPs/TIMP1 ratio observed in S-Exo-treated wounds closely mirrors scarless fetal wound healing patterns (Dang et al., 2003), underscoring the therapeutic potential of S-Exo for dynamic ECM remodeling and improved scar outcomes. Similar beneficial ECM remodeling effects have been reported with adipose-derived mesenchymal stem cell exosomes, further supporting the clinical translational potential of exosome-based therapies (Wang et al., 2017).

Our study showed that S-Exo treatment led to significant metabolic reprogramming in fibroblasts, prominently enhancing glycolysis, the TCA cycle, and the pentose phosphate pathway (PPP) (Figures 4–7). Specifically, S-Exo increased glucose consumption, glycolytic intermediates, and anabolic fluxes, supporting energy metabolism, macromolecule synthesis, and antioxidant capacity, thereby creating an optimal microenvironment for tissue repair. The potential of exosomes to transfer key molecules, including metabolic enzymes, to regulate cellular metabolism has been increasingly recognized (Isaac et al., 2021). Previous studies have demonstrated that exosomes mediate metabolic reprogramming (Yang et al., 2020). Exosomes derived from mesenchymal stem cells induced by advanced glycation end products (AGEs) have been shown to reprogram endothelial cell metabolism, shifting their energy metabolism toward glycolysis, which affects cell survival and angiogenesis, further highlighting the role of exosomes in metabolic modulation during tissue repair (Liang et al., 2025). Tubular epithelial cell-derived extracellular vesicles have been reported to induce macrophage glycolysis by stabilizing HIF-1α in diabetic kidney disease (Jia et al., 2022). Recent studies also emphasize the critical role of glycolysis in maintaining stemness and supporting the proliferation of stem cells. Metabolic modulation—particularly enhanced glycolytic flux—has thus been proposed as a promising strategy to regulate stem cell fate and function. In line with this, exosomes derived from skeletal muscle were shown to transfer glycolytic enzymes (e.g., LDHA, PKM, UGP2, ENO3) to bone marrow mesenchymal stem cells, promoting aerobic glycolysis and osteogenic differentiation (Ma S. et al., 2023). Our findings further support this notion, revealing significant enrichment of glycolytic enzymes in S-Exo (Table 7), which likely contributes to the observed metabolic reprogramming in fibroblasts. Moreover, we observed increased expression of glycolytic genes (GLUT1, PFKM, HK2) and enhanced metabolic flux in recipient fibroblasts, indicating coordinated regulation of cellular metabolism. This enzyme transfer, coupled with endogenous induction, may represent a novel exosome-mediated mechanism, warranting further mechanistic investigation. However, whether these exosome-delivered enzymes retain their functional activity within recipient cells remains to be clarified and will be the focus of future investigation.

Glycolytic inhibition using 2-DG significantly reduced S-Exo-induced MMP1 and MMP3 secretion, indicating that glycolysis may contribute to ECM remodeling (Figure 6). One possible mechanism is that enhanced glycolysis increases the availability of metabolic intermediates such as acetyl-CoA, a critical substrate for histone acetyltransferases (Shen et al., 2015). Studies have shown that glycolysis-mediated changes in acetyl-CoA levels play a key role in regulating chromatin modifications and gene expression (Wapenaar et al., 2016). In embryonic stem cells, glycolysis provides the necessary acetyl-CoA for histone acetyltransferase, and downregulation of glycolytic flux during differentiation leads to decreased acetyl-CoA levels, resulting in histone deacetylation and silencing of pluripotency genes (Moussaie et al., 2015). Elevated acetyl-CoA production promotes the acetylation of histones, relaxing chromatin structure and facilitating the transcription of MMP genes, which may contribute to their increased secretion during tissue repair (Yeh et al., 2016; Qin and Han, 2010; Ibarrola et al., 2024). Alternatively, activation of AMP-activated protein kinase (AMPK), a central regulator of cellular energy homeostasis known to respond to shifts in glycolytic flux, may directly or indirectly modulate MMP expression through downstream signaling pathways (Ong et al., 2015; Wang et al., 2022). Our proteomic analyses revealed the enrichment of the AMPK pathway (Figure 8), which provides further insights into its potential role in regulating ECM remodeling. Further mechanistic studies are necessary to fully elucidate this relationship, potentially revealing novel therapeutic targets connecting energy metabolism to ECM remodeling.

Our proteomic analysis revealed significant overlaps between S-Exos and previously reported exosomes from human minor salivary gland organoids (Qian et al., 2024), particularly in critical pathways such as glycolysis/gluconeogenesis, the PPP, PI3K-Akt signaling, and ECM–receptor interactions (Figure 8). These shared pathways are integral to metabolic regulation, cell growth signaling, and extracellular matrix dynamics, suggesting a conserved proteomic composition inherent to salivary exosomes. Such consistency underscores the inherent therapeutic potential of saliva-derived exosomes in tissue regeneration and wound repair. However, this study also has limitations. Although multi-omics analyses provided a comprehensive understanding of metabolic reprogramming mediated by S-Exos, the precise molecular mechanisms by which exosomal cargoes interact with recipient cells require further exploration.

## Conclusion

In summary, our findings suggest that saliva-derived exosomes may significantly enhance skin wound healing by modulating fibroblast metabolism. Specifically, our data indicate that these exosomes contain most enzymes involved in glycolysis, potentially driving sustained glycolytic flux and promoting the expression of key glycolytic enzymes such as HK2 and PFKM in recipient fibroblasts. Moreover, we observed a positive correlation between increased glycolytic activity and elevated MMP secretion, suggesting a metabolic link to ECM remodeling.

## Data Availability

The datasets presented in this study can be found in online repositories. The names of the repository/repositories and accession number(s) can be found below: https://www.ncbi.nlm.nih.gov/, PRJNA1203059.
