# Supplementary figures and images for: Saliva-derived exosomes regulate fibroblast metabolic reprogramming in skin wound healing
Source: Front Cell Dev Biol. 2025 Jul 23;13:1606716. doi: 10.3389/fcell.2025.1606716 (PMC12325227; doi:10.3389/fcell.2025.1606716)

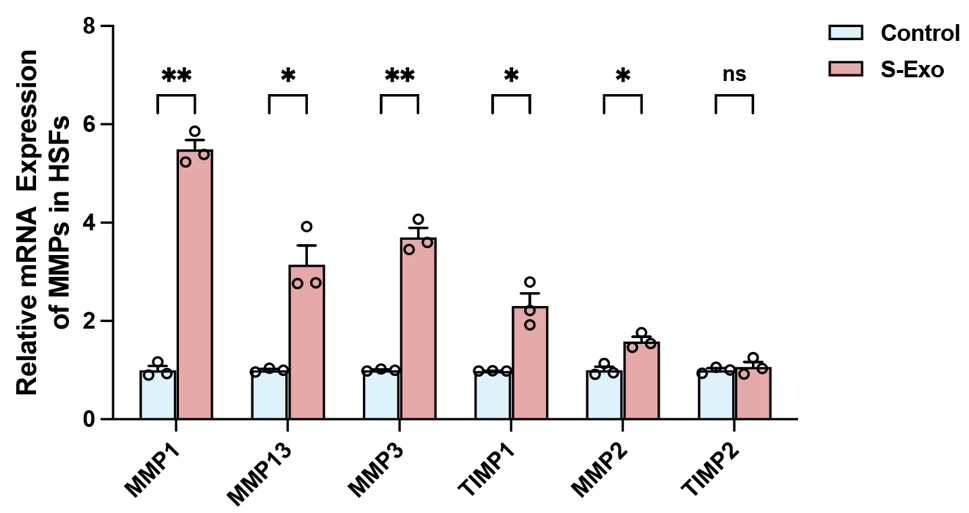


Figure S1: RT-qPCR analysis of MMPs and TIMPs mRNA expression changes in HSFs.

Supplement: Supplementary file 1 [file DataSheet1.docx]
